# Cytoprotective effect of flavonoid‐induced autophagy on bisphosphonate mediated cell death in osteoblast

**DOI:** 10.1002/jcb.26728

**Published:** 2018-03-12

**Authors:** Jung‐Han Kim, Hae‐Mi Kang, Su‐Bin Yu, Jae‐Min Song, Chul‐Hoon Kim, Bok‐Joo Kim, Bong‐Soo Park, Sang‐Hun Shin, In‐Ryoung Kim

**Affiliations:** ^1^ Department of Oral and Maxillofacial Surgery Pusan National University Dental Hospital Yangsan‐si Gyeongsangnam‐do South Korea; ^2^ Department of Oral and Maxillofacial Surgery Medical center, Dong‐A University, Seo‐gu Busan South Korea; ^3^ Department of Oral Anatomy School of Dentistry Pusan National University, Busandaehak‐ro Yangsan‐si Gyeongsangnam‐do South Korea; ^4^ BK21 PLUS Project School of Dentistry Pusan National University, Busandaehak‐ro Yangsan‐si Gyeongsangnam‐do South Korea

**Keywords:** autophagy, bisphosphonate, flavonoid, osteoblast, osteogenesis, zoledronate

## Abstract

With rapid economic growth and further developments in medical science, the entry into the aging population is currently increasing, as is the number of patients with metabolic diseases, such as hypertension, hyperlipidemia, heart disease, and diabetes. The current treatments for metabolic bone diseases, which are also on the rise, cause negative side effects. Bisphosphonates, which are used to treat osteoporosis, inhibit the bone resorption ability of osteoclasts and during prolonged administration, cause bisphosphonate‐related osteonecrosis of the jaw (BRONJ). Numerous studies have shown the potential role of natural plant products as flavonoids in the protection against osteoporosis and in the influence of bone remodeling. Autophagy occurs after the degradation of cytoplasmic components within the lysosome and serves as an essential cytoprotective response to pathologic stress caused by certain diseases. In the present study, we hypothesized that the cytoprotective effects of flavonoids might be related to those associated with autophagy, an essential cytoprotective response to the pathologic stress caused by certain diseases, in osteoblasts. We demonstrated the cytoprotective effect of flavonoid‐induced autophagy against the toxicity of zoledronate and the induction of autophagy by flavonoids to support osteogenic transcription factors, leading to osteoblast differentiation and bone formation. Further studies are necessary to clarify the connections between autophagy and osteogenesis. It would be helpful to shed light on methodological challenges through molecular biological studies and new animal models. The findings of the current study may help to delineate the potential role of flavonoids in the treatment of metabolic bone disease.

## 1 INTRODUCTION

Osteoporosis, a well‐known metabolic bone disease, is characterized by a low bone mass and the micro‐architectural degeneration of bone tissue.^1^, ^2^ The development of metabolic bone disease occurs through imbalances between the formation and resorption of bone by interactions between osteoblasts and osteoclasts.^3^ Imbalances of remodeling can result in normal structure and function, morbidity, and/or a shortened lifespan.^4^ Osteoporosis prevention and bone mass regeneration remain a challenge.^5^ Currently known treatments for osteoporosis include bisphosphonates, calcitonin, and estrogen; however, these drugs have relatively few therapeutic effects.^6^ Bisphosphonates have been widely used as the standard treatment for osteoporosis, Paget's disease, and metastatic bone disease.^7^ Bisphosphonates inhibit biochemical markers for bone resorption and promote osteoclast apoptosis, both in vitro and in vivo.^8^ Nitrogen‐containing bisphosphonates (N‐BP) predominantly act on osteoclasts; among N‐BP, zoledronate, exhibits the most potent pharmacological action.^9^, ^10^ For the last 30 years, N‐BP have been used clinically; therefore, awareness of their adverse reactions has also increased. Esophagitis, vasculitis, pyrexia, hypocalcemia, *hypophosphatemia*, and during long‐term treatment BP might be accumulate at the skeletal lesions it would be develop bisphosphonate‐related osteonecrosis of the jaw (BRONJ), however, the cause of side effects of the drug is not yet clear.^11^ Generally, bisphosphonates are known to stimulate osteoblast proliferation and differentiation and to inhibit apoptosis. However, recently, several studies showed that bisphosphonates, especially zoledronate, suppress osteoblastic production, and induce cytotoxicity toward osteoblasts in vitro.^11^, ^12^

Numerous studies have shown the potential role of natural plant products in protecting against osteoporosis and influencing bone remodeling.^13^, ^14^ In particular, flavonoids are a polyphenolic compound found in roots, leaves, vegetables, fruits, and plant beverages, and are well known for their cytoprotective effects, as well as their anti‐oxidant, anti‐inflammatory, anti‐cancer, and anti‐bacterial activities.^15^, ^16^, ^17^ Several flavonoids, such as diosmetin, chrysin, galangin, taxifolin, quercitrin, kaempferol, icariin, and quercetin, are known to have osteogenic and anti‐osteoclastogenic effects.^18^, ^19^, ^20^, ^21^

Autophagy, a major cellular‐degradative process, plays an important role in maintaining homeostasis, which requires protein degradation to produce energy; this is done by removing damaged substrates for recycling.^22^, ^23^ Under apoptotic stimuli, autophagy contributes to cellular protection by reducing the mitochondrial load in cells and thereby lowering the amount of proapoptotic molecules as cytochrome c is released from mitochondria.^22^ Recent studies reported that autophagy appeared to be involved in the differentiantion of osteoclasts, osteoblasts, and osteocytes, potentially pointing to a new pathogenic mechanism of bone homeostasis and bone marrow disease; however, this mechanism is still unclear.^24^ Therefore, we hypothesized that flavonoids promote osteogenic differentiation and cytoprotective effects by inducing autophagy against zoledronate‐induced osteoblast apoptosis.

In the present study, we investigated the effects of zoledronate on the viability, functions, and osteogenetic and cytoprotective effects of flavonoids, including galangin, icariside II, kaempferol, and quercetin, in human fetal osteoblastic (hFOB) 1.19 cells. Furthermore, we investigated whether the cytoprotective effects of these flavonoids were related to autophagy.

## 2 MATERIALS AND METHODS

### 2.1 Reagents

**Figure 1:**
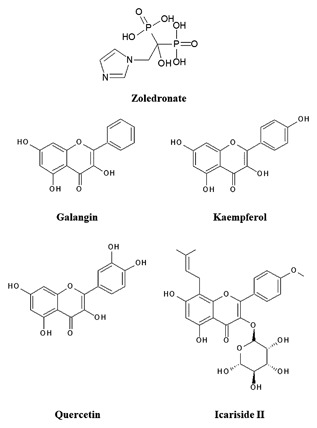
Chemical structures of zoledronate and various flavonoids

Zoledronate, galangin, icariside II, kaempferol, quercetin, Zoledronate, galangin, icariside II, kaempferol, quercetin (Figure 1), and 3‐[4,5‐dimethylthiazol‐2‐yl]‐2,5‐diphenyl tetrazolium bromide reagents were purchased from Sigma (St. Louis, MO), and 3‐methyladenine (3‐MA; a type‐III phosphatidylinositol 3‐kinase [PI‐3 K] inhibitor) and rapamycin reagents were obtained from Calbiochem (La Jolla, CA). The antibodies against beclin‐1 and Autophagy protein 5 (ATG5) were purchased from Cell Signaling Technology (Beverly, MA). The antibodies against collagen I, osteocalcin, bone morphogenetic protein 2 (BMP‐2), Osterix, and RUNX2 were purchased from ABCam (Cambridge, MA). The antibodies against LC3 were purchased from Sigma. SQSTM1/p62, β‐actin antibody, mouse anti‐rabbit IgG antibody, and rabbit anti‐mouse IgG antibody were purchased from Santa Cruz Biotechnology (Santa Cruz, CA). All other chemicals and reagents were purchased from Sigma unless otherwise specified.

### 2.2 Cell culture and differentiation

The hFOB 1.19 cells used in this study were purchased from the American Type Culture Collection (Rockville, MD). The cells were cultured in a DMEM/F12 1:1 medium with a 10% fetal bovine serum and 1% penicillin‐streptomycin (Hyclone, GE Healthcare Life Sciences, Logan, UT) at 37°C. To induce differentiation, we used the culture method described in.^25^

### 2.3 Treatment with zoledronate and flavonoids

Zoledronate (100 mM) was dissolved in DMSO and stored at −20°C until use. Before the zoledronate treatment, hFOB 1.19 cells were grown to about 80‐90% confluence and then exposed to zoledronate at different concentrations (0‐100 µM) for at least 24 h and up to 14 days in a humidified atmosphere of 5% CO_2_ at 37°C. The flavonoids (ie, galangin, icariside II, kaempferol, and quercetin) were also dissolved in DMSO and were treated with various concentrations of flavonoids (0‐50 µM) for at least 24 h up to 72 h.

### 2.4 MTT assay

The cytotoxicity of zoledronate and the flavonoids (ie, galangin, icariside II, kaempferol, and quercetin) was tested using a tetrazolium dye (MTT) assay, as described in.^25^ The hFOB 1.19 cells were treated with zoledronate, galangin, icariside II, kaempferol, and quercetin at various concentrations (0‐100 µM) for 24‐72 h. Cell viability was measured on an ELISA reader (Sunrise Remote Control, Tecan, Austria) at an excitation emission wavelength of 570 nm.

### 2.5 Morphological analysis of cells using confocal microscopy

The hFOB 1.19 cells (1 × 10^4^) were seeded on a Lab‐Tek™ II Chamber Slide (Nunc; Thermo Fisher Scientific, Rochester, NY) for 24 h. The next day, the cells were treated with galangin (5 µM), autophagy inhibitor 3‐MA (500 µM), and autophagy inducer rapamycin (50 nM) for 24 h. CYTO‐ID® Green autophagy dye was used to detect autophagy in live cells.^26^ The autophagosomes and autolysosomes were stained using a CYTO‐ID® Autophagy Detection Kit (Enzo Life Sciences, Farmingdale, NY) and analyzed using confocal microscopy (Carl Zeiss, Germany). Staining was conducted following manufacturer’ protocol.

### 2.6 Alkaline phosphatase (ALP) activity

The hFOB 1.19 cells were plated in 24‐well plate at a density of 2 × 10^5^ cells per well. Following treatment with zoledronate and flavonoids, the cells were incubated for at least 3 days and up to 14 days in a 5% CO_2_ incubator at 37°C. The cell extraction was conducted as described in.^5^ ALP activity was measured using a Leukocyte Alkaline Phosphatase Kit (Sigma). A standard curve was created using p‐nitrophenol as the standard, and each value was normalized to the protein concentration. The ALP activity of each sample was also normalized to the protein concentration and measured by an ELISA reader at 405 nm. ALP staining was also conducted using a Leukocyte Alkaline Phosphatase Kit (Sigma). The staining was conducted following the manufacturer's protocol. The stained cells were then photographed under an inverted microscope.

### 2.7 Alizarin Red S staining

Alizarin Red S staining was performed to visualize the mineralization of the extracellular matrix. The hFOB 1.19 cells were plated in 24‐well plates at a density of 2 × 10^5^ cells/well. Following the treatment with zoledronate and flavonoids, the cells were incubated for 14 days. Then, the cells were washed with PBS and fixed with 4% paraformaldehyde for 15 min. The cells were stained with 2% Alizarin Red S solution (pH 4.2) for 10 min and washed carefully twice with tap water. The stained cells were then photographed under an inverted microscope. The quantification of Alizarin Red S was conducted as described in a previous study.^27^ The absorbance of the cells was measured at 550 nm using an ELISA reader (Sunrise Remote Control, Tecan, Austria).

### 2.8 Western blot assays

The hFOB 1.19 (1 × 10^6^) cells were plated in 100‐mm culture dishes. The next day, the cells were treated with various reagents as necessary for the purpose of each experiment. The cells were harvested and washed twice with ice‐cold PBS. The total cell proteins were lysed with an radioimmunoprecipitation assay (RIPA) buffer (Invitrogen) at 4°C for 1 h. Protein lysis and immunoblotting were conducted using previously described methods.^28^ Immunostaining with antibodies was performed using a SuperSignal West femto‐enhanced chemiluminescence substrate and detected using an AlphaImager HP (Alpha Innotech, Santa Clara, CA). Equivalent protein loading was confirmed by Ponceau S staining.

### 2.9 Real‐time PCR

Total cellular RNA was isolated from the hFOB 1.19 cells using an RNeasy Mini Kit (Qiagen, Hilden, Germany) according to the manufacturer's instructions. The total RNA (2 µg) was reverse‐transcribed using the M‐MLV cDNA Synthesis Kit (Enzynomics, Daejeon, Korea) according to the manufacturer's protocol, for real‐time PCR; real‐time PCR was performed on an ABI 7500 Fast Real‐Time PCR System (Applied Biosystems 7500 Sequence Detection System, version 2.6.1) using TOPreal™ qPCR 2X PreMIX (SYBR Green, Enzynomics, Daejeon, Korea). The running conditions were as described previously in.^5^ The primers used in this study are shown in Table 1.

**Table 1:** Primers used in this study

Gene	Forward primer (5′‐3′)	Reverse primer (5′‐3′)
Collagen I	TGAACTTGTTGCTGAGGGCA	ACTGGGCCAATGTCCACAAA
BMP‐2	GCTGTCTTCTAGCGTTGCTG	CTGTTTCAGGCCGAACATGC
ALP	ATCTTCCTGGGTGACGGGAT	CATGGCCAGGAAGGTCTCAG
RUNX‐2	GCGCATTCCTCATCCCAGTA	GGCTCAGGTAGGAGGGGTAA
Osterix	AAACCCAAGGCAGTGGGAAA	TGCCCCCATATCCACCACTA
LC3B	GGCCTTCTTCCTGTTGGTGA	TCTCCTGGGAGGCATAGACC
SQSTM1	CATCGGAGGATCCGAGTGTG	TTCTTTTCCCTCCGTGCTCC
GAPDH	GTCAAGGCTGAGAACGGGAA	AAATGAGCCCCAGCCTTCTC

### 2.10 Statistical analysis

All the experiments were performed in triplicate (*n *= 3), and the results were displayed as mean ± SD. Statistical analyses were performed using GraphPad Prism 5.0 for Windows (GraphPad Software, San Diego, CA). In the statistical analysis, a one‐way ANOVA was used for Dunnett's multiple‐comparison test.

## 3 RESULTS

### 3.1 Zoledronate inhibits cell viability and induces apoptosis in hFOB 1.19 cells

**Figure 2:**
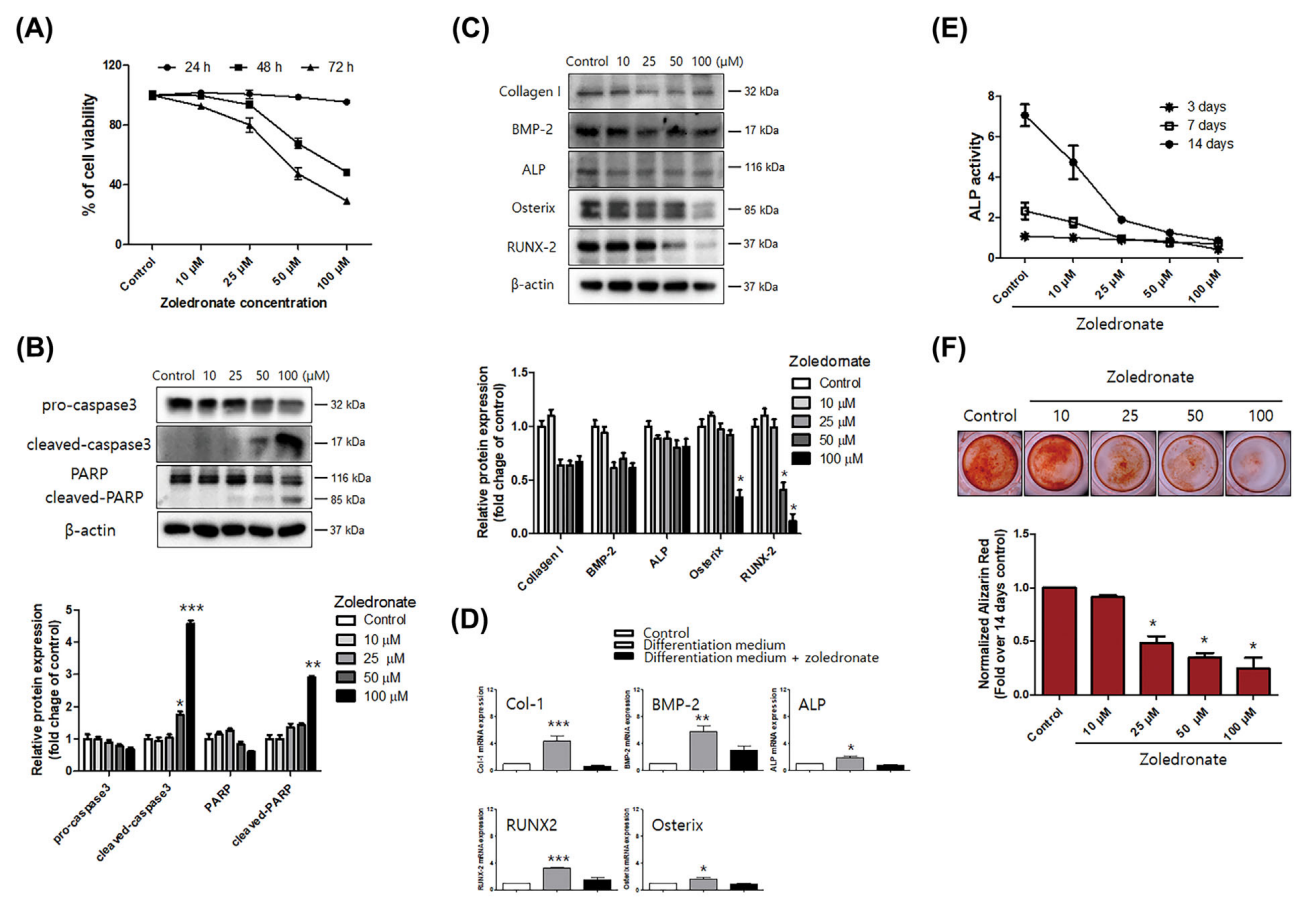
Zoledronate activates apoptosis‐related proteins and inhibits osteoblast differentiation in hFOB 1.19 cells. A, The cell viability of zoledronate‐treated (10‐100 µM) cells incubated for 24‐72 h was determined with an MTT assay. B, Zoledronate (10‐100 µM) was incubated for 48 h. The expression of apoptosis‐related proteins, including pro‐caspase‐3, cleaved caspase‐3, and PARP, was determined using Western blot analysis. β‐actin was used as an internal control. C, Western blot analysis and (D) real‐time PCR showed that the osteogenesis‐associated factors of collagen I, BMP‐2, ALP, Osterix, and RUNX2 were remarkably inhibited in a dose‐dependent manner by zoledronate treatment. E, After zoledronate treatment, the cells were incubated for 3, 7, and 14 days, and the ALP activity was measured using an ELISA reader. F, Alizarin Red S staining to determine osteoblast mineralization was measured using an ELISA reader. For the Alizarin Red S staining, the zoledronate‐treated cells were incubated for 14 days. The data are expressed as mean ± SD (*n* = 3) and were analyzed by one‐way ANOVA using Dunnett's multiple‐comparison test. (*P < 0.05, ** *P* < 0.01, and ***P < 0.001 for the difference between the control group and each treatment group)

To determine the cell viability of zoledronate, an hFOB 1.19 cell, we examined a wide concentration range of zoledronate (0‐100 µM) in these cells by an MTT assay. The cells were treated with zoledronate and incubated for 24‐72 h. There was no change in cell viability in the cells treated with zoledronate (0‐100 µM) at 24 h. After 48 h of zoledronate treatment with concentrations over 25 µM cell viability significantly reduced dose dependently, to 80.1% (25 µM), 47.4% (50 µM), and 29.3% (100 µM) at 48 h, and 93.6% (25 µM), 67.7% (50 µM), and 48.5% (100 µM) at 72 h (Figure 2A), respectively. When death stimuli affects the cells, they undergo apoptosis by caspase activation and nuclear apoptotic events.^29^ Zoledronate treatment also showed the activation of pro‐caspase‐3 and the cleavage products of caspase‐3 and poly ADP ribose polymerase (PARP) in hFOB 1.19 cells, as detected using Western blot assays (Figure 2B). These results indicate that zoledronate has cytotoxicity and induces apoptosis in hFOB 1.19 cells.

### 3.2 Zoledronate inhibits cell differentiation and mineralization in hFOB 1.19 cells

To further investigate the alteration of osteogenesis‐associated factors by zoledronate, the cells were treated with various concentrations of zoledronate (0‐100 µM) for 24 h. Differentiation‐inducing media, including ascorbic acid and β‐glycerophosphate, were used, along with Western blot assays and real‐time PCR. Zoledronate remarkably inhibited the expression of osteogenesis‐associated proteins (Figure 2C), including collagen I, BMP‐2, ALP, Osterix, and RUNX2. The mRNA levels of collagen I, BMP‐2, ALP, Osterix, and RUNX2 were remarkably inhibited in the zoledronate‐treated group (Figure 2D).

To determine the inhibitory effects of differentiation and mineralization by zoledronate, an ALP activity assay and Alizarin Red S staining were performed. ALP is a membrane‐bound enzyme, and its activity can be used as an initial indicator of osteoblast differentiation.^30^ To evaluate ALP activity, the hFOB 1.19 cells were treated with zoledronate (0‐100 µM) and incubated for 3‐14 days. The ALP activity reduced significantly in accordance with the increase in the concentration of zoledronate after 7 days (Figure 2E). Alizarin Red S is a dye that selectively binds calcium salts, and it is used to detect, and quantify mineralization.^31^ Hence, the calcified cells appeared bright red in color after Alizarin Red S staining (Figure 2F). Zoledronate also inhibited the calcification of hFOB 1.19 cells dose dependently. As zoledronate significantly reduced ALP activity, the intensity of Alizarin Red S staining, and the mRNA and/or protein levels of the osteogenic markers, we conclude that zoledronate inhibits osteoblastic differentiation and mineralization and that it has osteogenic potential.

### 3.3 Flavonoids show cytoprotective effects against zoledronate‐induced cell damage

**Figure 3:**
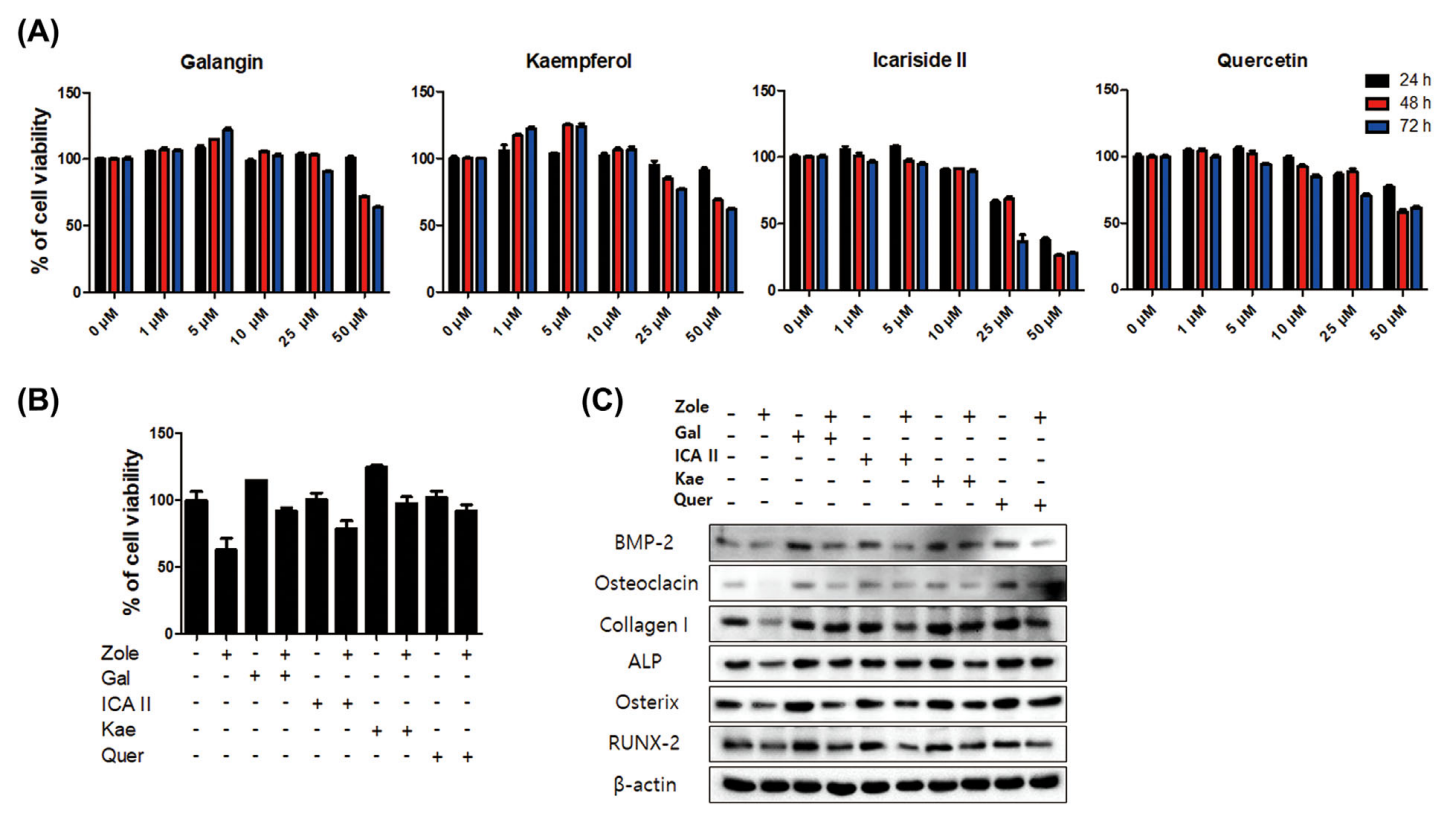
Flavonoids show cytoprotective effects on the hFOB 1.19 cells treated with zoledronate. A, The hFOB 1.19 cells were treated with galangin, icariside II, kaempferol, and quercetin (1‐50 µM) for 24‐72 h, and cell viability was assessed using an MTT assay, which was measured using an ELSIA reader. B, The hFOB 1.19 cells were pretreated with galangin (5 µM), icariside II (1 µM), kaempferol (5 µM), and quercetin (5 µM) for 24 h; after, they were treated with zoledronate (50 µM) for 48 h. The cells pretreated with flavonoids and treated with zoledronate showed higher viability than the cells treated with zoledronate alone. C, Western blot analysis showed that collagen I, osteocalcin, BMP‐2, ALP, Osterix, and RUNX2 increased in the cells pretreated with flavonoids and treated with zoledronate more than the cells treated with zoledronate alone

To confirm the cytotoxic effects of flavonoids on hFOB 1.19 cells, an MTT assay was used. The flavonoids used in the experiment were galangin, kaempferol, icariside II, and quercetin. These flavonoids have been reported to enhance osteoblastic differentiation and activity.^21^, ^32^, ^33^ According to our results, the flavonoids did not have high toxicity, and the survival rates of 5 µM treatment of galangin and kaempferol increased time dependently (Figure 3A).

The cytoprotective effects of flavonoids were confirmed using an MTT assay and Western blot analysis. The hFOB 1.19 cells were pretreated with galangin(5 µM), icariside II (1 µM), kaempferol (5 µM), and quercetin (5 µM) for 24 h; after, they were post‐treated with zoledronate (50 µM) for 48 h. The combined treatment group of flavonoids and zoledronate showed a much higher level of cell viability than the group treated with zoledronate alone (Figure 3B). The protein levels of collagen I, osteocalcin, BMP‐2, ALP, Osterix, and RUNX2 increased with the combined treatment (flavonoids and zoledronate) more than in the group that was just treated with zoledronate (Figure 3C).

**Figure 4:**
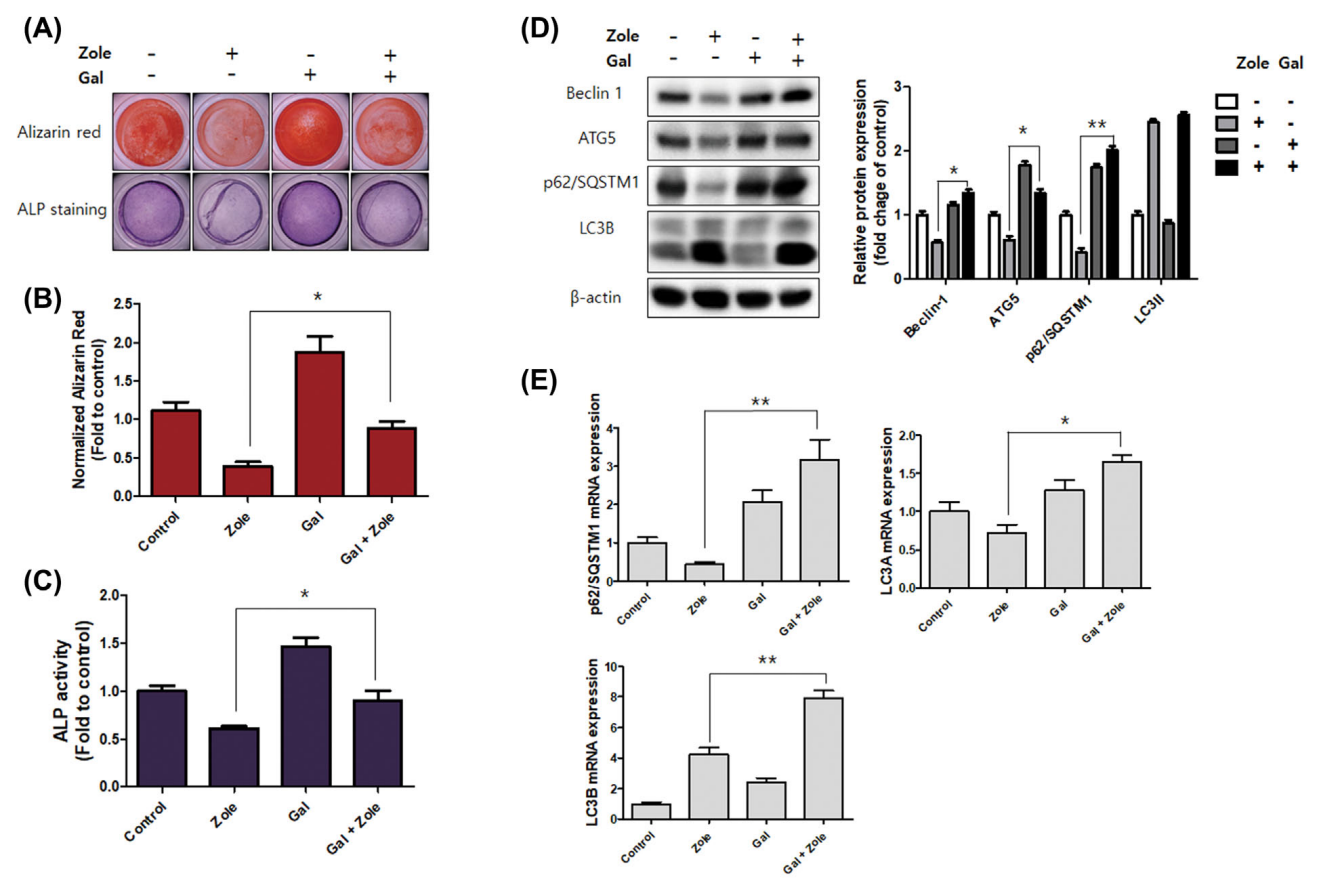
Galangin activates osteoblast differentiation and autophagy‐related factors. A‐C, The hFOB 1.19 cells were pretreated with galangin (5 µM) for 24 h, then treated with zoledronate (50 µM) for 48 h; after treatment, the cells were incubated for 14 days in differentiation‐inducing media. A, Alizarin Red S (upper panel) and ALP staining (lower panel) were conducted to find changes in mineralization and differentiation. The graph indicates the quantification of Alizarin Red S staining (B) and ALP activity (C). D and E, The hFOB 1.19 cells were pretreated with galangin (5 µM) for 24 h, then treated with zoledronate (50 µM) for 48 h. D, Western blotting used antibodies specific for beclin‐1, ATG5, SQSTM1/p62, LC3, and β‐actin. β‐actin was used as an internal control. E, The gene expressions of SQSTM1/p62, LC3A, and LC3B were analyzed by real‐time PCR. The data are expressed as mean ± SD (*n* = 3) and were analyzed by one‐way ANOVA using Dunnett's multiple‐comparison test. (*P < 0.05 and **P < 0.01 for the difference between the zoledronate and Galangin + zoledronate group)

We performed the following experiments with galangin, which showed the most effective cytoprotection and the highest expression levels of osteogenic markers among the flavonoids The hFOB 1.19 cells were pretreated with galangin (5 µM) for 24 h, then post‐treated with zoledronate (50 µM) for 48 h. Following the treatment, the cells induced differentiation and mineralization. Alizarin Red S staining and the ALP activity assay showed a much higher measured value for the combined treatment with flavonoids and zoledronate than they did for the zoledronate treatment (Figure 4A‐C).

### 3.4 Galangin induces autophagy and is associated with the differentiation of osteoblasts

Recent studies have reported possible mechanisms by which autophagy mediates cytoprotective effects.^22^ In the present study, we hypothesized that the cytoprotective effects of flavonoids might be related to the cytoprotective effects associated with autophagy Thus, to further investigate the induction of autophagy in osteoblasts by galangin treatment, we analyzed a combined treatment of flavonoids with zoledronate, and compared it with a treatment using only zoledronate. As in previous experiments, the hFOB 1.19 cells were pretreated with galangin (5 µM) for 24 h, then treated with zoledronate (50 µM) for 48 h. The expression levels of beclin‐1, ATG5, SQSTM1/p62, and LC3A were reduced when the cells were just treated with zoledronate, but these levels increased with a galangin treatment and with a combined treatment of galangin and zoledronate (Figures 4D and 4E).

**Figure 5:**
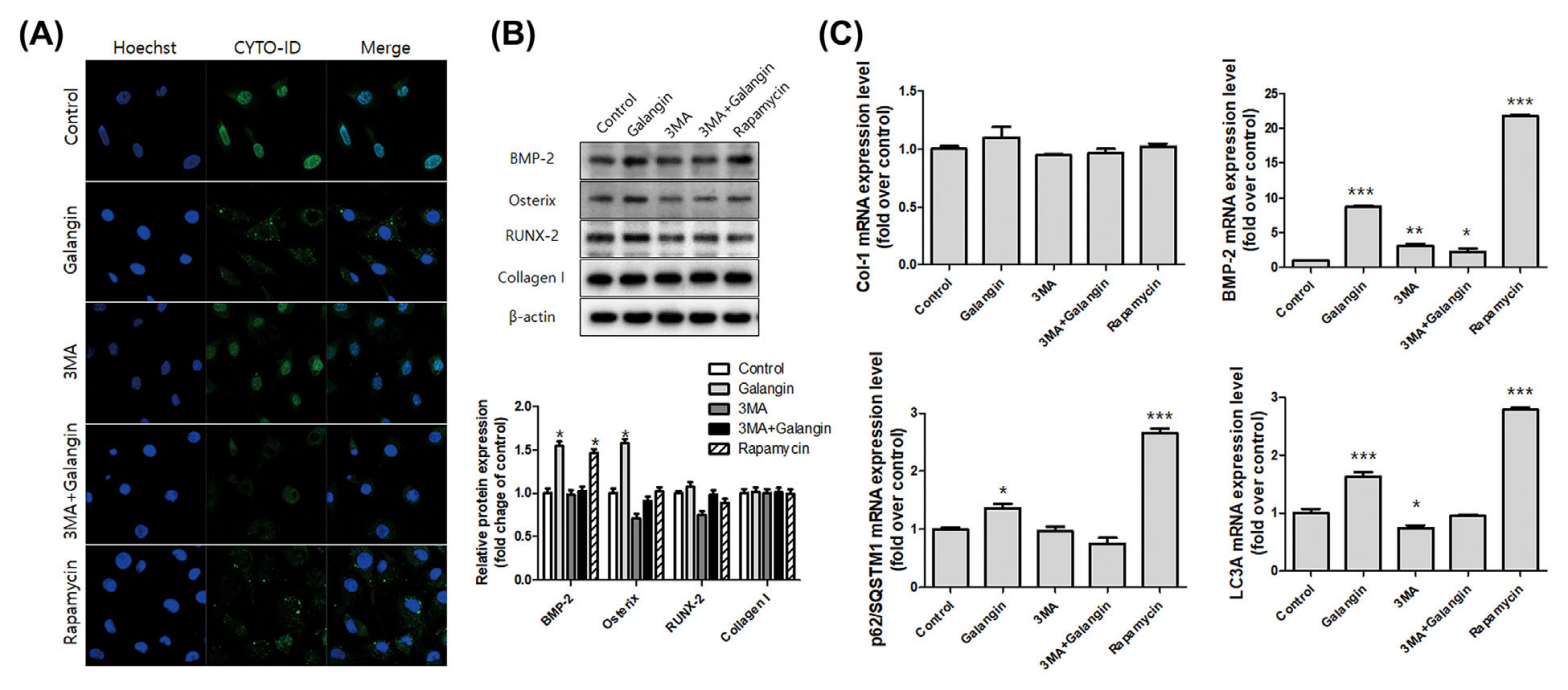
Galangin induces autophagy and activates osteogenesis‐associated factors. A, The cells were grown on a lab‐tag chamber slide and treated with galangin (5 µM), autophagy inhibitor 3‐MA (500 µM), and autophagy inducer rapamycin (50 nM) for 24 h. The autophagosomes and autolysosomes were stained with a CYTO‐ID® Autophagy Detection Kit and analyzed by confocal microscopy. B, The expression levels of collagen I, osteocalcin, BMP‐2, ALP, Osterix, and RUNX2 were assessed using Western blot analysis. C, The gene expressions of collagen I, BMP‐2, SQSTM1/p62, and LC3A were analyzed by real‐time PCR. The data are expressed as mean ± SD (*n* = 3) and was analyzed by one‐way ANOVA using Dunnett's multiple‐comparison test. (*P < 0.05, **P < 0.01, and ***P < 0.001 for the difference between the control group and each treatment group)

We next evaluated the activation of autophagy in the osteoblasts induced by galangin and confirmed the formation of autophagosomes and autolysosomes using confocal microscopy. It is known that rapamycin induces autophagy via the inhibition of the mammalian target of rapamycin and that 3‐MA blocks the formation of autophagosomes.^34^ Thus, the cells were treated with galangin (5 µM), autophagy inhibitor 3‐MA (500 µM), and autophagy inducer rapamycin (50 nM) for 24 h. Rapamycin was used as a positive control for autophagy in the hFOB 1.19 cells in this experiment. The treatment with galangin induced the formation of autophagosomes in the hFOB 1.19 cells; conversely, the combination treatment with 3‐MA, and galangin did not induce autophagosome formation (Figure 5A). Finally, we hypothesized that galangin‐induced autophagy regulates osteoblast differentiation and bone formation. Thus, following these results, the combined treatment of 3‐MA and galangin blocked the induction of osteoblast differentiation markers, such as collagen I, BMP‐2, Osterix, and RUNX2, more than a treatment of galangin alone (Figure 5B); in addition, the gene expressions of Collagen I, BMP‐2, SQSTM1/p62, and LC3A were inhibited by the combined treatment of 3‐MA and galangin (Figure 5C). These results clearly indicate that galangin induces autophagy and that autophagy activates osteoblast differentiation.

## 4 DISCUSSION

Bisphosphonates are commonly prescribed to postmenopausal women to treat osteoporosis. Osteoporosis treatment aims to inhibit the resorption of trabecular bones by osteoclasts, thus preserving bone density.^35^ Pamidronate, zoledronate, and alendronate have been linked to BRONJ.^36^ Generally, the therapeutic efficacy of bisphosphonates in osteoporosis is known to be caused, in part, by its ability to prevent osteocyte, and osteoblast apoptosis.^37^ Recently, several studies have shown that bisphosphonates suppress the growth, differentiation, and antigenic profiles of osteoblasts by altering their physiology, which would explain the disruption of their repair capacity and may be directly related to the development of BRONJ.^11^, ^12^, ^38^ Therefore, in the present study, we used hFOB 1.19 cells to determine whether zoledronate induces apoptosis and inhibits osteoblastic differentiation and mineralization (Figure 2).

Flavonoids are naturally occurring bioactive compounds found in fruits and plants, and they have been linked to several health benefits, including anti‐oxidative, free‐radical scavenging, coronary heart disease preventative, and anti‐cancer effects in humans and animals.^39^, ^40^, ^41^ Previous studies have revealed that flavonoids exhibit cytoprotective effects in injured cells through direct anti‐oxidative activity.^42^, ^43^, ^44^ In this experiment, we used galangin, icariside II, kaempferol, and quercetin, since they are known to promote osteoblast differentiation.^21^, ^32^, ^33^ These flavonoids were shown to increase the proliferation of hFOB 1.19 cells at low doses. Pre‐incubation with galangin (5 µM), icariside II (1 µM), kaempferol (5 µM), and quercetin (5 µM) significantly prevents zoledronate‐induced cell damage; in particular, galangin and kaempferol showed high cell protection against zoledronic stress. In addition, in the osteogenic process, BMP induces osteogenic signaling pathways.^45^

RUNX2 and the RUNX2‐targeted gene Osterix are master transcription factors of osteogenesis, and they are known to promote the expression of ALP, osteocalcin, osteopontin, osteonectin, and collagen I.^45^, ^46^ In this study, zoledronate was shown to remarkably inhibit the expression of BMP‐2, Osterix, and RUNX2 (Figure 2). Pre‐incubation with flavonoids that have cytoprotective effects was shown to protect gene expression and support the differentiation of osteoblasts against zoledronic damage (Figure 3).

Autophagy occurs through the degradation of cytoplasmic components within the lysosome^47^ and serves as an essential cytoprotective response to pathologic stress that are caused by diseases like cancer, ischemia, and infection.^48^ In the present study, we hypothesized that the cytoprotective effects of flavonoids might be related to the cytoprotective effects associated with autophagy. Autophagy plays an essential quality control function in the cell by promoting the basal turnover of long‐lived proteins and organelles as well as by selectively degrading damaged cellular components.^22^, ^48^ The expression levels of beclin‐1, ATG5, SQSTM1/p62, and LC3A are reduced when cells are treated with zoledronate alone, are increased with a galangin treatment, and are increased with a combined treatment of galangin and zoledronate.

LC3s (LC3A, B, and C), a key component of autophagy, are structural proteins in autophagosomal membranes. LC3A is localized in perinuclear and nuclear regions, while LC3B is equally distributed throughout the cytoplasm and nucleolar regions. LC3C is extensively co‐localized with LC3A and beclin‐1 in the cytoplasm and nucleolar regions. Autophagosomes are formed by only one of the three LC3 proteins in the cytoplasm.^49^ Nihira et al^50^ reported that its possible functions could include autophagosomal formation and maturation, which is not compensated by LC3B, as LC3A is co‐localized with autophagosomes. Our data showed that LC3B expression was not involved in galangin‐induced autophagy; however, LC3A showed high mRNA expression. Therefore, we can conclude that galangin induces autophagy, which shows cytoprotective activity, and that LC3A, not LC3B, is involved in autophagosome formation.

We hypothesized that galangin‐induced autophagy might be involved in osteoblast differentiation and bone formation. 3‐MA is known to block the formation of autophagosomes^34^ and inhibit the formation of autophagosomes by galangin treatment; it also blocks the expression of osteogenic transcription factors, such as BMP‐2, Osterix, RUNX2, and collagen I (Figure 5). These results clearly supported our hypotheses that galangin induces autophagy and that autophagy activates osteoblast differentiation.

The present study demonstrated the cytoprotective effect of flavonoid‐induced autophagy against the toxicity of zoledronate. The induction of autophagy by flavonoids can be used to support osteogenic transcription factors, which leads to osteoblast differentiation and bone formation. Further studies are necessary to clarify the connection between autophagy and osteogenesis. Molecular biological studies and new animal models will help to shed light on methodological challenges in this area. The findings of the current study may help to delineate the potential role of flavonoids as an alternative to bisphosphonate, proposing further research on flavonoids as a therapeutic agent for metabolic bone disease.

## CONFLICTS OF INTEREST

The authors declare that there are no conflicts of interest.
